# The one-dimensional organic–inorganic hybrid: *catena*-poly[bis­[1-(3-ammonio­prop­yl)-1*H*-imidazolium] [[iodidoplumbate(II)]-tri-μ-iodido-plumbate(II)-tri-μ-iodido-[iodidoplumbate(II)]-di-μ-iodido]]

**DOI:** 10.1107/S160053681100941X

**Published:** 2011-03-15

**Authors:** A. Trigui, H. Boughzala, A. Driss, Y. Abid

**Affiliations:** a Laboratoire de Physique Appliquée (LPA), Faculté des Sciences de Sfax, 3018, BP 802, Tunisia; b Laboratoire de Cristallochimie et des Matériaux, Faculté des Sciences de Tunis, Tunisia

## Abstract

The organic–inorganic hybrid, {(C_6_H_13_N_3_)_2_[Pb_3_I_10_]}_*n*_, was obtained by the reaction of 1-(3-ammonio­prop­yl)imidazolium triiodide and PbI_2_ at room temperature. The structure contains one-dimensional {[Pb_3_I_10_]^4−^}_*n*_ polymeric anions spreading parallel to [001], resulting from face–face–edge association of PbI_6_ distorted octa­hedra. One of the Pb^II^ cations is imposed at an inversion centre, whereas the second occupies a general position. N—H⋯I hydrogen bonds connect the organic cations and inorganic anions.

## Related literature

For organic–inorganic hybrid materials, see: Billing & Lemmerer (2004 ▶); Dammak *et al.* (2009 ▶); Elleuch *et al.* (2007 ▶, 2010 ▶); Gebauer & Schmid (1999 ▶); Ishihara *et al.* (1990 ▶); Krautscheid *et al.* (2001 ▶). For the structures of lead iodide-based complexes, see: Maxcy *et al.* (2003 ▶); Mitzi *et al.* (2001 ▶); Mousdis *et al.* (1998 ▶); Papavassiliou *et al.* (1999 ▶); Samet Kallel *et al.* (2008 ▶).
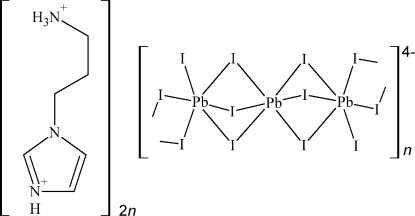


## Experimental

### 

#### Crystal data

(C_6_H_13_N_3_)_2_[Pb_3_I_10_]
*M* *_r_* = 2144.99
Triclinic, 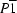

*a* = 8.652 (3) Å
*b* = 11.728 (5) Å
*c* = 11.972 (6) Å
α = 117.21 (3)°
β = 98.05 (2)°
γ = 107.17 (3)°
*V* = 976.7 (9) Å^3^
*Z* = 1
Mo *K*α radiation
μ = 20.81 mm^−1^
*T* = 293 K
0.40 × 0.20 × 0.02 mm

#### Data collection

Enraf–Nonius CAD-4 diffractometer
Absorption correction: ψ scan (North *et al.*, 1968 ▶) *T*_min_ = 0.139, *T*_max_ = 0.624
4950 measured reflections
3796 independent reflections
2749 reflections with *I* > 2σ(*I*)
*R*_int_ = 0.024
2 standard reflections every 120 min  intensity decay: 6%

#### Refinement

*R*[*F* ^2^ > 2σ(*F* ^2^)] = 0.036
*wR*(*F* ^2^) = 0.110
*S* = 1.02
3796 reflections
143 parameters
H-atom parameters constrained
Δρ_max_ = 2.17 e Å^−3^
Δρ_min_ = −2.09 e Å^−3^

### 

Data collection: *CAD-4 EXPRESS* (Duisenberg, 1992 ▶); cell refinement: *CAD-4 EXPRESS*; data reduction: *XCAD4* (Harms & Wocadlo, 1995 ▶); program(s) used to solve structure: *SHELXS97* (Sheldrick, 2008 ▶); program(s) used to refine structure: *SHELXL97* (Sheldrick, 2008 ▶); molecular graphics: *DIAMOND* (Brandenburg, 2006 ▶); software used to prepare material for publication: *publCIF* (Westrip, 2010 ▶).

## Supplementary Material

Crystal structure: contains datablocks I, global. DOI: 10.1107/S160053681100941X/yk2002sup1.cif

Structure factors: contains datablocks I. DOI: 10.1107/S160053681100941X/yk2002Isup2.hkl

Additional supplementary materials:  crystallographic information; 3D view; checkCIF report

## Figures and Tables

**Table 1:** Selected geometric parameters (Å, °)

Pb1—I5	3.155 (2)
Pb1—I1	3.1757 (13)
Pb1—I3	3.2264 (14)
Pb1—I1^i^	3.2652 (13)
Pb1—I2	3.3039 (14)
Pb1—I4	3.309 (2)
Pb2—I4	3.2105 (15)
Pb2—I3	3.2388 (14)
Pb2—I2	3.263 (2)

Symmetry code: (i) 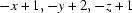.

**Table 2:** Hydrogen-bond geometry (Å, °)

*D*—H⋯*A*	*D*—H	H⋯*A*	*D*⋯*A*	*D*—H⋯*A*
N9—H9*A*⋯I5	0.89	2.84	3.67 (2)	156
N9—H9*B*⋯I2^iii^	0.89	2.90	3.68 (2)	147
N9—H9*C*⋯I5^iv^	0.89	2.89	3.64 (2)	143

Symmetry codes: (iii) 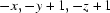; (iv) 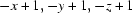.

**Fig. 1.**
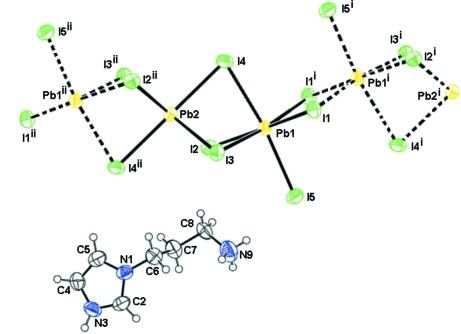
View of the asymmetric unit of (C6H13N3)2Pb3I10 with some adjacent atoms showing the atom-labelling scheme. Displacement ellipsoids are drawn at the 50% probability level. [Symmetry codes: (i) 1 - x, 2 - y, 1 - z, (ii) 1 - x, 2 - y, 2 - z]

**Fig. 2.**
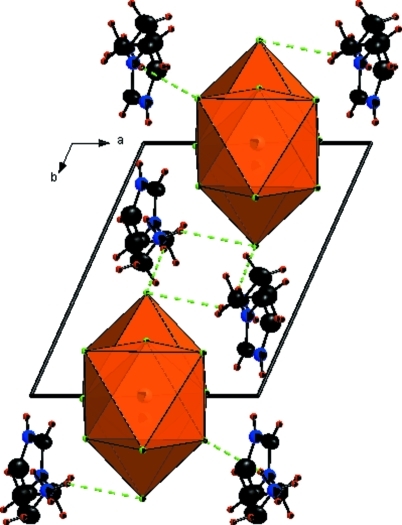
The crystal packing of (C6H13N3)2Pb3I10 viewed along [001] direction and showing the N—H···I hydrogen bonding (dashed lines).

## supplementary crystallographic information

### Comment

Recently, self-assembling organic–inorganic hybrid compounds have been the focus of a great number of investigations owing to their unique structural, magnetic, optical nonlinear and optoelectronic functionality (Papavassiliou *et al.*, 1999; Ishihara *et al.*, 1990; Mitzi *et al.*, 2001). In particular, the lead iodide-based hybrid materials have been extensively studied (Gebauer & Schmid, 1999; Dammak *et al.*, 2009; Elleuch *et al.*, 2010) since they show strong room temperature excitonic optical features with large exciton binding energy and oscillator strengths. These low dimensional complexes include zero dimensional (0D), one dimensional (1D), and two dimensional (2D) lead iodide networks with organic groups as spacers. Among them, 1D-hybrids are more attractive in nanoscaled applications since they form a variety of crystalline structures, which differ in the inorganic chain where the [PbI_6_] octahedra can be connected in different ways: face sharing (Elleuch *et al.*, 2007), edge sharing (Samet Kallel *et al.*, 2008), corner sharing (Mousdis *et al.*, 1998) or through several combinations of these various types of sharing (Maxcy *et al.*, 2003; Billing & Lemmerer, 2004; Krautscheid *et al.*, 2001), as in the case of our compound. We present here the structure of the organic–inorganic one dimensional hybrid compound (C_6_H_13_N_3_)_2_Pb_3_I_10_.

The crystal structure of the title compound consists of (Pb_3_I_10_)_n_^4n-^ chains extending along [001] with the 1-(3-ammoniopropyl)-imidazolium cations as counter-ions (Fig. 1). The inorganic anion, shown in Fig. 1, can be considered as a set of mixed face-shared/edge-shared octahedra. In fact, the unit cell contains three octahedra with two crystallographically independent Pb atoms: Pb1 and Pb2. The central Pb2 octahedron is connected to the Pb1 octahedra by shared faces, while the Pb1 octahedra are linked *via* edge-sharing at both ends of  Pb_3_I_10_^4-^ to adjacent units.

The coordination octahedron of the central lead ion Pb2 is only slightly distorted since it is located on an inversion centre and is bound to three unique I atoms: I2, I3 and I4, which participate in the face-sharing between the Pb2 and Pb1 octahedra. The bond lengths around Pb2 are very similar (3.2105 (15), 3.2388 (14), 3.263 (2) Å), the bond angles I—Pb2—I deviate slightly from ideal octahedral values, ranging from 83° to 94°. In contrast, Pb1 has a more distorted environement with Pb—I distances ranging from 3.155 (2) to 3.309 (2) Å and with all *cis* and *trans* angles different (see Table 1). This Pb atom is bonded to five unique I atoms, where two I1 atoms are responsible for the edge sharing between the neighbouring units to form one-dimensional infinite chains. Atom I5 is the only halide not involved in any bonding with adjacent octahedra and has the shortest Pb—I distance [3.155 (2) Å].

Cations fill channels between the anionic chains (Fig.2). Each terminal ammonium group forms three N—H···I hydrogen bonds to I atoms of three different chains (see Table 2).

### Experimental

Single crystals of (C_6_H_13_N_3_)_2_Pb_3_I_10_ were grown by the slow evaporation at room temperature of a solution containing PbI_2_ and C_6_H_13_N_3_I_3_ salts. An aqueous solution of HI was added to the aminopropylimidazole to synthesize C_6_H_13_N_3_I_3_ precursor. Under ambient conditions, stoechiometric amounts of C_6_H_13_N_3_I_3_ and PbI_2_ with excess HI were sailed in DMF. This mixture was stirred and remained clear without any precipitate. Pale-yellow flatted crystals were obtained few weeks later. Supplementary data for this paper are available from the IUCr electronic archives (Reference: CCDC 782074).

### Refinement

All H atoms attached to C and N atom were fixed geometrically and treated as riding with C—H = 0.97 Å (CH_2_) or 0.93 Å (CH) and N—H = 0.89 Å (NH_3_) or 0.86 Å (NH) with *U*_iso_(H) = 1.2U_eq_(C or N).

### Crystal data

**Table:**

(C_6_H_13_N_3_)_2_[Pb_3_I_10_]	*Z* = 1
*M**_r_* = 2144.99	*F*(000) = 916
Triclinic, *P*1	*D*_x_ = 3.647 Mg m^−^^3^
Hall symbol: -P 1	Mo *K*α radiation, λ = 0.71073 Å
*a* = 8.652 (3) Å	Cell parameters from 25 reflections
*b* = 11.728 (5) Å	θ = 9–15°
*c* = 11.972 (6) Å	µ = 20.81 mm^−^^1^
α = 117.21 (3)°	*T* = 293 K
β = 98.05 (2)°	Flat, yellow
γ = 107.17 (3)°	0.4 × 0.2 × 0.02 mm
*V* = 976.7 (9) Å^3^	

### Data collection

**Table:**

Enraf–Nonius CAD-4 diffractometer	2749 reflections with *I* > 2σ(*I*)
Radiation source: fine-focus sealed tube	*R*_int_ = 0.024
graphite	θ_max_ = 26.0°, θ_min_ = 2.0°
non–profiled ω/2θ scans	*h* = −10→2
Absorption correction: ψ scan (North *et al.*, 1968)	*k* = −14→14
*T*_min_ = 0.139, *T*_max_ = 0.624	*l* = −14→14
4950 measured reflections	2 standard reflections every 120 min
3796 independent reflections	intensity decay: 6%

### Refinement

**Table:**

Refinement on *F*^2^	Secondary atom site location: difference Fourier map
Least-squares matrix: full	Hydrogen site location: inferred from neighbouring sites
*R*[*F*^2^ > 2σ(*F*^2^)] = 0.036	H-atom parameters constrained
*wR*(*F*^2^) = 0.110	*w* = 1/[σ^2^(*F*_o_^2^) + (0.063*P*)^2^] where *P* = (*F*_o_^2^ + 2*F*_c_^2^)/3
*S* = 1.01	(Δ/σ)_max_ = 0.001
3796 reflections	Δρ_max_ = 2.17 e Å^−^^3^
143 parameters	Δρ_min_ = −2.09 e Å^−^^3^
0 restraints	Extinction correction: *SHELXL97* (Sheldrick, 2008), Fc^*^=kFc[1+0.001xFc^2^λ^3^/sin(2θ)]^-1/4^
Primary atom site location: structure-invariant direct methods	Extinction coefficient: 0.00169 (17)

### Special details

**Table:**

Geometry. All e.s.d.'s (except the e.s.d. in the dihedral angle between two l.s. planes) are estimated using the full covariance matrix. The cell e.s.d.'s are taken into account individually in the estimation of e.s.d.'s in distances, angles and torsion angles; correlations between e.s.d.'s in cell parameters are only used when they are defined by crystal symmetry. An approximate (isotropic) treatment of cell e.s.d.'s is used for estimating e.s.d.'s involving l.s. planes.
Refinement. Refinement of *F*^2^ against ALL reflections. The weighted *R*-factor *wR* and goodness of fit *S* are based on *F*^2^, conventional *R*-factors *R* are based on *F*, with *F* set to zero for negative *F*^2^. The threshold expression of *F*^2^ > σ(*F*^2^) is used only for calculating *R*-factors(gt) *etc*. and is not relevant to the choice of reflections for refinement. *R*-factors based on *F*^2^ are statistically about twice as large as those based on *F*, and *R*-factors based on ALL data will be even larger.

### Fractional atomic coordinates and isotropic or equivalent isotropic displacement parameters (Å^2^)

**Table:**

	*x*	*y*	*z*	*U*_iso_*/*U*_eq_	
Pb1	0.45091 (5)	0.90517 (4)	0.62320 (4)	0.03669 (15)	
Pb2	0.5000	1.0000	1.0000	0.03708 (18)	
I1	0.76440 (9)	0.98779 (8)	0.52450 (7)	0.0436 (2)	
I2	0.14853 (9)	0.82298 (7)	0.74775 (7)	0.0436 (2)	
I3	0.67368 (10)	0.82982 (8)	0.79737 (7)	0.0432 (2)	
I4	0.59957 (11)	1.21598 (7)	0.90100 (8)	0.0505 (2)	
I5	0.30697 (11)	0.59287 (8)	0.38118 (8)	0.0501 (2)	
N9	0.2562 (18)	0.3757 (14)	0.5290 (12)	0.073 (4)	
H9A	0.2959	0.4198	0.4885	0.109*	
H9B	0.1595	0.3006	0.4732	0.109*	
H9C	0.3332	0.3487	0.5545	0.109*	
C8	0.223 (2)	0.4705 (14)	0.6451 (14)	0.060 (4)	
H8A	0.1398	0.5000	0.6172	0.073*	
H8B	0.3281	0.5534	0.7047	0.073*	
C7	0.1565 (18)	0.3985 (15)	0.7187 (14)	0.057 (3)	
H7A	0.1032	0.4516	0.7762	0.069*	
H7B	0.0682	0.3058	0.6536	0.069*	
C6	0.2864 (16)	0.3825 (13)	0.8013 (12)	0.050 (3)	
H6A	0.3363	0.3245	0.7449	0.060*	
H6B	0.3773	0.4739	0.8663	0.060*	
N1	0.2042 (13)	0.3173 (9)	0.8694 (9)	0.045 (2)	
C2	0.1429 (17)	0.1818 (12)	0.8230 (12)	0.050 (3)	
H2	0.1488	0.1127	0.7458	0.060*	
N3	0.0710 (14)	0.1625 (10)	0.9077 (11)	0.054 (3)	
H3	0.0212	0.0825	0.8993	0.065*	
C4	0.0878 (19)	0.2879 (14)	1.0096 (14)	0.058 (3)	
H4	0.0471	0.3025	1.0808	0.069*	
C5	0.1731 (18)	0.3842 (14)	0.9870 (13)	0.057 (3)	
H5	0.2065	0.4805	1.0411	0.069*	

### Atomic displacement parameters (Å^2^)

**Table:**

	*U*^11^	*U*^22^	*U*^33^	*U*^12^	*U*^13^	*U*^23^
Pb1	0.0386 (3)	0.0395 (2)	0.0362 (2)	0.01781 (19)	0.01491 (18)	0.02124 (19)
Pb2	0.0412 (3)	0.0372 (3)	0.0332 (3)	0.0167 (3)	0.0139 (2)	0.0179 (2)
I1	0.0373 (4)	0.0564 (4)	0.0468 (4)	0.0216 (4)	0.0182 (3)	0.0318 (4)
I2	0.0343 (4)	0.0428 (4)	0.0479 (4)	0.0126 (3)	0.0151 (3)	0.0210 (3)
I3	0.0473 (4)	0.0506 (4)	0.0447 (4)	0.0300 (4)	0.0203 (3)	0.0272 (3)
I4	0.0590 (5)	0.0321 (4)	0.0514 (4)	0.0121 (4)	0.0103 (4)	0.0213 (3)
I5	0.0543 (5)	0.0409 (4)	0.0411 (4)	0.0177 (4)	0.0087 (4)	0.0140 (3)
N9	0.090 (10)	0.089 (9)	0.074 (8)	0.051 (8)	0.035 (7)	0.057 (7)
C8	0.085 (11)	0.058 (8)	0.062 (8)	0.034 (8)	0.029 (7)	0.045 (7)
C7	0.063 (9)	0.065 (8)	0.062 (8)	0.030 (7)	0.026 (7)	0.042 (7)
C6	0.045 (7)	0.053 (7)	0.055 (7)	0.021 (6)	0.019 (6)	0.030 (6)
N1	0.044 (6)	0.038 (5)	0.042 (5)	0.015 (4)	0.006 (4)	0.017 (4)
C2	0.055 (8)	0.038 (6)	0.041 (6)	0.021 (6)	0.005 (6)	0.012 (5)
N3	0.056 (7)	0.041 (5)	0.058 (6)	0.007 (5)	0.009 (5)	0.030 (5)
C4	0.068 (9)	0.061 (8)	0.058 (8)	0.026 (7)	0.038 (7)	0.037 (7)
C5	0.064 (9)	0.045 (7)	0.051 (7)	0.020 (7)	0.018 (7)	0.018 (6)

### Geometric parameters (Å, °)

**Table:**

Pb1—I5	3.155 (2)	C8—H8A	0.9700
Pb1—I1	3.1757 (13)	C8—H8B	0.9700
Pb1—I3	3.2264 (14)	C7—C6	1.502 (18)
Pb1—I1^i^	3.2652 (13)	C7—H7A	0.9700
Pb1—I2	3.3039 (14)	C7—H7B	0.9700
Pb1—I4	3.309 (2)	C6—N1	1.473 (16)
Pb2—I4	3.2105 (15)	C6—H6A	0.9700
Pb2—I4^ii^	3.2105 (14)	C6—H6B	0.9700
Pb2—I3	3.2388 (14)	N1—C2	1.315 (15)
Pb2—I3^ii^	3.2388 (14)	N1—C5	1.375 (16)
Pb2—I2^ii^	3.263 (2)	C2—N3	1.331 (17)
Pb2—I2	3.263 (2)	C2—H2	0.9300
I1—Pb1^i^	3.2653 (13)	N3—C4	1.362 (16)
N9—C8	1.460 (17)	N3—H3	0.8600
N9—H9A	0.8900	C4—C5	1.318 (19)
N9—H9B	0.8900	C4—H4	0.9300
N9—H9C	0.8900	C5—H5	0.9300
C8—C7	1.536 (19)		
			
I5—Pb1—I1	90.09 (5)	C8—N9—H9C	109.5
I5—Pb1—I3	89.89 (5)	H9A—N9—H9C	109.5
I1—Pb1—I3	88.94 (4)	H9B—N9—H9C	109.5
I5—Pb1—I1^i^	95.96 (5)	N9—C8—C7	111.1 (11)
I1—Pb1—I1^i^	92.18 (4)	N9—C8—H8A	109.4
I3—Pb1—I1^i^	174.05 (2)	C7—C8—H8A	109.4
I5—Pb1—I2	91.62 (5)	N9—C8—H8B	109.4
I1—Pb1—I2	175.04 (2)	C7—C8—H8B	109.4
I3—Pb1—I2	86.41 (4)	H8A—C8—H8B	108.0
I1^i^—Pb1—I2	92.27 (4)	C6—C7—C8	116.4 (12)
I5—Pb1—I4	172.82 (3)	C6—C7—H7A	108.2
I1—Pb1—I4	94.09 (5)	C8—C7—H7A	108.2
I3—Pb1—I4	84.36 (5)	C6—C7—H7B	108.2
I1^i^—Pb1—I4	89.73 (5)	C8—C7—H7B	108.2
I2—Pb1—I4	83.75 (5)	H7A—C7—H7B	107.3
I4—Pb2—I4^ii^	180.0	N1—C6—C7	109.8 (10)
I4—Pb2—I3	85.76 (4)	N1—C6—H6A	109.7
I4^ii^—Pb2—I3	94.24 (4)	C7—C6—H6A	109.7
I3—Pb2—I3^ii^	180.0	N1—C6—H6B	109.7
I4^ii^—Pb2—I2	94.02 (5)	C7—C6—H6B	109.7
I4—Pb2—I2	85.98 (5)	H6A—C6—H6B	108.2
I3^ii^—Pb2—I2	93.10 (5)	C2—N1—C5	108.9 (12)
I3—Pb2—I2	86.90 (5)	C2—N1—C6	124.3 (10)
I2^ii^—Pb2—I2	180.0	C5—N1—C6	126.8 (10)
I3^ii^—Pb2—I2^ii^	86.90 (5)	N1—C2—N3	106.8 (10)
I3—Pb2—I2^ii^	93.10 (5)	N1—C2—H2	126.6
I4^ii^—Pb2—I2^ii^	85.98 (5)	N3—C2—H2	126.6
I4—Pb2—I2^ii^	94.02 (5)	C2—N3—C4	110.1 (10)
I4—Pb2—I3^ii^	94.24 (4)	C2—N3—H3	124.9
I4^ii^—Pb2—I3^ii^	85.76 (4)	C4—N3—H3	124.9
Pb1—I1—Pb1^i^	87.82 (4)	C5—C4—N3	106.2 (11)
Pb2—I2—Pb1	76.05 (4)	C5—C4—H4	126.9
Pb1—I3—Pb2	77.46 (4)	N3—C4—H4	126.9
Pb2—I4—Pb1	76.68 (5)	C4—C5—N1	108.0 (12)
C8—N9—H9A	109.5	C4—C5—H5	126.0
C8—N9—H9B	109.5	N1—C5—H5	126.0
H9A—N9—H9B	109.5		

Symmetry codes: (i) −*x*+1, −*y*+2, −*z*+1; (ii) −*x*+1, −*y*+2, −*z*+2.

### Hydrogen-bond geometry (Å, °)

**Table:**

*D*—H···*A*	*D*—H	H···*A*	*D*···*A*	*D*—H···*A*
N9—H9A···I5	0.89	2.84	3.67 (2)	156
N9—H9B···I2^iii^	0.89	2.90	3.68 (2)	147
N9—H9C···I5^iv^	0.89	2.89	3.64 (2)	143

Symmetry codes:  (iii) −*x*, −*y*+1, −*z*+1; (iv) −*x*+1, −*y*+1, −*z*+1.
